# Direct Reprogramming of Human Fibroblasts to Hepatocyte-Like Cells by Synthetic Modified mRNAs

**DOI:** 10.1371/journal.pone.0100134

**Published:** 2014-06-25

**Authors:** Kamen P. Simeonov, Hirdesh Uppal

**Affiliations:** Department of Investigative Toxicology, Genentech, South San Francisco, California, United States of America; Institute of Medical Biology, Singapore

## Abstract

Direct reprogramming by overexpression of defined transcription factors is a promising new method of deriving useful but rare cell types from readily available ones. While the method presents numerous advantages over induced pluripotent stem (iPS) cell approaches, a focus on murine conversions and a reliance on retroviral vectors limit potential human applications. Here we address these concerns by demonstrating direct conversion of human fibroblasts to hepatocyte-like cells via repeated transfection with synthetic modified mRNAs. Hepatic induction was achieved with as little as three transcription factor mRNAs encoding HNF1A plus any two of the factors, FOXA1, FOXA3, or HNF4A in the presence of an optimized hepatic growth medium. We show that the absolute necessity of exogenous *HNF1A* mRNA delivery is explained both by the factor's inability to be activated by any other factors screened and its simultaneous ability to strongly induce expression of other master hepatic transcription factors. Further analysis of factor interaction showed that a series of robust cross-activations exist between factors that induce a hepatocyte-like state. Transcriptome and small RNA sequencing during conversion toward hepatocyte-like cells revealed global preferential activation of liver genes and miRNAs over those associated with other endodermal tissues, as well as downregulation of fibroblast-associated genes. Induced hepatocyte-like cells also exhibited hepatic morphology and protein expression. Our data provide insight into the process by which direct hepatic reprogramming occurs in human cells. More importantly, by demonstrating that it is possible to achieve direct reprogramming without the use of retroviral gene delivery, our results supply a crucial step toward realizing the potential of direct reprogramming in regenerative medicine.

## Introduction

Direct reprogramming or conversion, where one cell type is directly converted into another without passage through a pluripotent intermediate, is an attractive source for valuable but unavailable cells, such as hepatocytes [1]. From basic and pharmaceutical research to cell therapy and regenerative medicine, cells derived by direct reprogramming offer near limitless potential [2]–[4]. Compared to more established methods of cellular derivation, such as embryonic or induced pluripotent stem cell (iPS) directed differentiation, direct reprogramming presents several advantages: lack of tumorigenic risk [5], fast conversion rate [6], and repair of injured tissues by *in vivo* reprogramming [7], [8]. However, two major issues prevent the field from reaching full potential: First, while a variety of conversions have been discovered in mouse models [9]–[13], most encounter difficulty when applied to human cells, likely due to the differences in the transcriptional circuits controlling reprogramming in human and mouse [6]. Second, all conversions have been performed by delivery of reprogramming factors and reporters using retroviral vectors [6], which integrate into the genome, often causing oncogenic transformations that prohibit downstream clinical applications [14]. Over the last eight years, the iPS field has successfully addressed similar issues. For the field of direct reprogramming to reach full scientific and particularly clinical relevance, these two issues must also be resolved. We address the first of these problems by investigating the factors required to convert human neonatal fibroblasts to a hepatic fate and the second by relying on synthetic modified mRNAs to overexpress reprogramming factors without genomic modification.

## Results

### Synthetic modified mRNAs and hepatic reprogramming media

**Figure 1:**
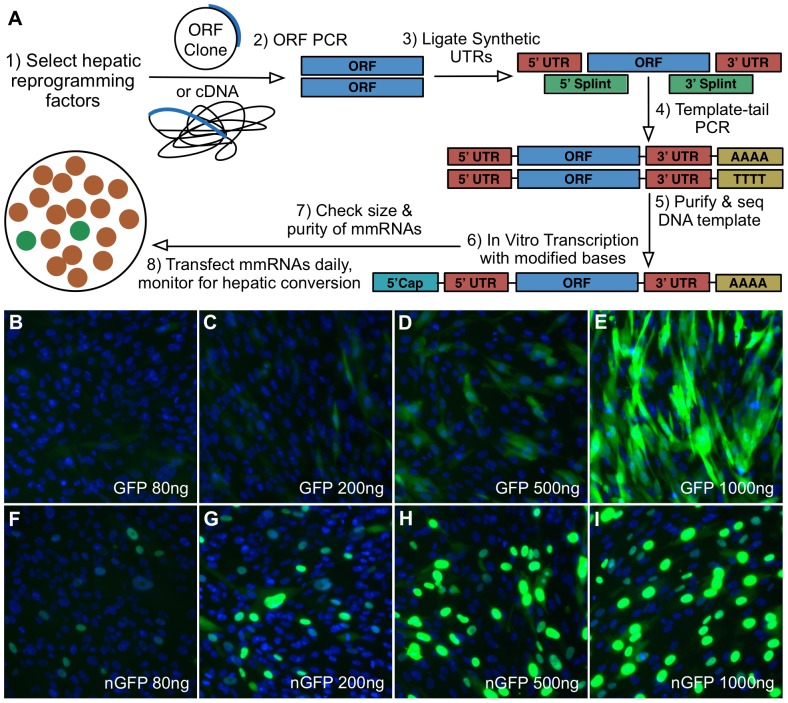
Production and transfection of synthetic modified mRNAs. (A) Scheme for producing DNA templates with synthetic UTR and PolyA sequences attached to the ORFs of interest. Primers used in the ORF PCR are gene specific. Primers used in template-tail PCR are independent of the gene and are always the same. Tailed-templates can be generated, purified, and used for overnight *in vitro* transcription in under two hours of bench time using this method. (B–I) Concentration-dependent translation and proper localization of GFP and nuclear GFP mmRNA at 80 ng, 200 ng, 500 ng, and 1000 ng per well in a 12-well plate. Nuclei are stained blue by Hoechst.

Eleven transcription factors (11TF) (Table 1), central to liver development [15]–[17], were selected as potential hepatic reprogramming factors. To overexpress factors without risk of genomic integration or modification, we generated synthetic modified mRNAs (mmRNAs) for each factor. We then pooled these at a one to one molar ratio to create the 11TF mix. These mmRNAs include synthetic base pair analogs and other modifications that maximize the RNA half-life, while minimizing cytotoxicity by limiting the cellular immune response against foreign RNA [18]. As the factors for reprogramming directly toward a hepatic fate are unknown in human, we required a quick and dynamic method of mmRNA generation. Traditional methods of mmRNA production were lengthy and inflexible for our needs as they were originally designed for reprogramming toward pluripotency [18], where the necessary factors have been long-established. Hence, we developed a rapid, exclusively PCR-based production scheme that allowed complete sequence-confirmed and quality-controlled mmRNAs to be generated in under four hours of bench time (Figure 1A). Transfection of mmRNAs encoding GFP (Figure 1B-1E) and nuclear GFP (nGFP) (Figure 1F-1I) into human neonatal fibroblasts demonstrated concentration dependent translation and proper localization. As previously reported [18], high levels of reprogramming genes could be maintained by daily transfection of mmRNAs over many days (Figure S1A) with no effect on cell viability (data not shown). Reprogramming factors were translated at appropriate levels and localized properly to the nucleus (Figure S1B-S1G)). We identified an optimal reprogramming media (Table S1) by screening an array of growth factors, small molecules, basal medias, and culture dish coatings for the ability to activate hepatic genes in human CD34+ bone marrow cells (data not shown), which have weak hepatic transdifferentiation potential [19], [20].

**Table 1:** Transcription factors used for hepatic reprogramming.

Gene Name	Accession Number
*CEBPA*	NM_004364
*GATA6*	NM_005257
*HHEX*	NM_002729
*HNF1B*	NM_000458
*HNF6A*	NM_004498
*FOXA2* *	NM_021784
*GATA4* *	NM_002052
*FOXA1* * 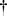	NM_004496
*FOXA3* * 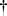	NM_004497
*HNF4A* * 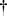	NM_000457
*HNF1A* * 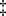	NM_000545

11TF includes all genes.

*Included in 6TF.

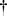
Sufficient for hepatic reprogramming when combined with *HNF1A*.

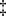
Necessary for hepatic reprogramming.

### Induction of hepatocyte-like cells by defined transcription factors

**Figure 2:**
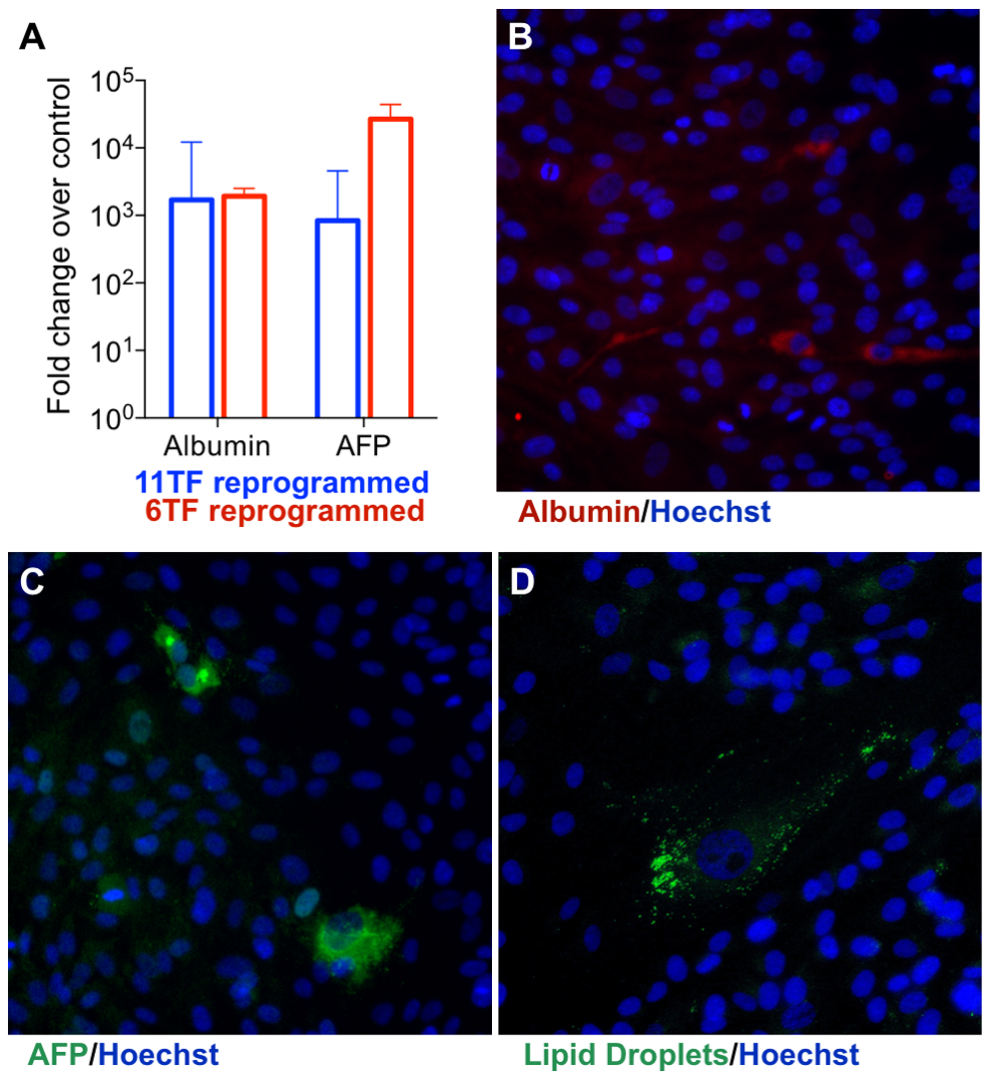
Hepatocyte-like state induced by 11TF and 6TF within five days. (A) Massive induction of hepatic genes, *ALB* and *AFP*, within five days of reprogramming (mean
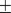
SD). Distinct albumin (B), AFP (C), and neutral lipid droplet (D) positive cells also appear within 5 days. Nuclei are stained blue by Hoechst.

Human neonatal fibroblasts were transferred to reprogramming media and transfected daily for five days with either 11TF mmRNA cocktail or empty vehicle control. To maintain cells free of genomic modification, we did not monitor for conversion using retroviral reporters, but instead directly measured the expression of hepatocyte-specific genes, albumin (*ALB*) (mature hepatocytes) and alpha-fetoprotein (*AFP*) (immature hepatocytes). Strikingly within five days of reprogramming, both genes were induced thousands-fold above vehicle control (n = 3) (Figure 2A). Encouraged by this success, we narrowed down the cocktail of eleven factors and transfected a subset of six factors (6TF, Table 1) that we identified as high potential based on two recent reports of direct conversion to hepatocyte-like cells in mouse [9], [10]. Reprogramming with 6TF, we again observed massive induction of both *ALB* and *AFP* (n = 14) (Figure 2A). As 6TF appeared to activate *ALB* and *AFP* to a similar or greater extent than 11TF and required less transcription factors, we decided to focus primarily on 6TF moving forward.

The massive inductions of albumin and (*AFP*) were driven by the appearance of distinct albumin and AFP high-expressing cells at a frequency of approximately 1∶1000 to 1∶10,000 (Figure 2B–C) after five days of reprogramming. Cells appeared healthy and correctly localized Hoechst to the nucleus. In hepatocyte-like cells, albumin (Figure S2) and AFP (Figure S3) exclusively localized to the cytoplasm. AFP-positive hepatocyte-like cells displayed altered morphology, such as decreased elongation, compared to the surrounding fibroblasts (Figure S3). No staining was observed in cells transfected with vehicle alone. Cells with other hepatic features, such as characteristically circular nuclei and neutral lipid staining (Figure 2D), as well as binucleation (Figure S4A), also appeared within five days at a similar frequency. After brief reprogramming without subsequent enrichment, an array of hepatocyte-specific genes, ranging from secretory proteins such as *FABP1*, enzymes such as *CYP3A4*, cytoskeletal proteins such as *KRT18*, and transporters such as *ABCB1*, were upregulated to levels between those of vehicle control fibroblasts and primary adult hepatocytes (Figure S4B). The hepatocytes displayed 10–1000 fold higher gene expression for most markers of hepatic maturity than the reprogrammed samples, which contained a heterogenous mixture of hepatocyte-like cells and surrounding unconverted fibroblasts. This was consistent with our observation that albumin-positive hepatocyte-like cells appear at a frequency of 1∶1000 to 1∶10,000 after five days of reprogramming. *AFP* expression was elevated approximately 1000-fold or higher in reprogrammed samples compared to control fibroblasts. Notably, *AFP*, a specific marker for fetal liver, immature hepatocytes, and hepatic progenitors, was also approximately 100-fold higher in reprogrammed samples compared to hepatocytes, indicating that hepatocyte-like cells expressed markers of hepatic immaturity as well as maturity. Reprogramming of human fetal fibroblasts (Figure S4C) and embryonic stem cells (Figure S4D) displayed similar efficiencies as neonatal fibroblasts.

For fibroblasts, reprogramming media did not induce *ALB* or *AFP* expression on its own when compared with the unoptimized basal medium (Figure S5A). In fact, without concurrent transfection, reprogramming media-only samples had 2-fold lower *ALB* expression than basal media-only samples, an observation correlated with the health of the fibroblasts. However, reprogramming media appeared to be strikingly synergistic when coupled with daily transfection of the reprogramming cocktail 6TF. Fibroblasts grown in reprogramming media and transfected daily for five days with 6TF mmRNAs showed increases in *ALB* expression by several 1000-fold and increases in *AFP* expression by over 100,000-fold in some cases (Figure S5A). On the other hand, fibroblasts grown in basal media transfected in the same manner with 6TF, achieved *ALB* expression increases no higher than 50-fold and *AFP* increases no higher than 10-fold (Figure S5B). This indicated that for successful induction of a hepatocyte-like state both reprogramming media and 6TF mmRNAs were required. The synthetic glucocorticoid, dexamethasone, appeared to be a particularly integral component of the hepatic reprogramming media. Removal of dexamethasone from culture media resulted in a 100-fold decrease in hepatic gene induction by 6TF, and to a lesser extent by 11TF, indicating that glucocorticoid response element binding [21] was necessary for hepatic reprogramming (Figure S5C). Additionally, mmRNAs were sufficient to activate *TLR3*, which is critical to efficient reprogramming to pluripotency [22] and possibly to other fates, (Figure S5D). Further glucocorticoid or *TLR3* stimulation did not improve efficiency of conversion.

### Global sequencing analysis of reprogrammed cells

As the transcription factors of 6TF and 11TF are not exclusively expressed in liver but are also involved in the specification of other developmentally related, particularly endodermal tissues [23], we sought to understand the initial changes during reprogramming. To this end, we performed complete transcriptome and small RNA sequencing on cells reprogrammed for 5 days with 11TF, 6TF, or vehicle control without bias introduced by enrichment for hepatic cells. Globally, gene and small RNA expression was similar between 6TF and 11TF reprogrammed cells with 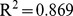 and 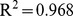 respectively. However, comparing 6TF reprogrammed cells versus vehicle control reprogrammed fibroblasts revealed that 6TF created a more diversified gene (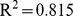) and small RNA (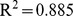) profile than 11TF created (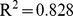 and 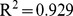, respectively) (Table 2). Based on these global results and our previous finding that 6TF was equal or superior to 11TF in *ALB* and *AFP* activation, we chose to continue focusing on 6TF for reprogramming.

**Table 2:** Global distribution of genes and small RNAs in 6TF, 11TF, and control.

	Number of Genes	Number of Small RNAs	R^2^ of Genes	R^2^ of Small RNAs
6TF vs. Control	18385	443	0.815	0.885
11TF vs. Control	18428	455	0.828	0.929
6TF vs. 11TF	18454	453	0.869	0.968

R^2^ values are calculated based on the log2 of the sequencing expression value for genes and small RNAs. The full expression data for all genes (Tables S4, S5, and S6) and small RNAs (Tables S7, S8, and S9) are provided in the supporting information.

**Figure 3:**
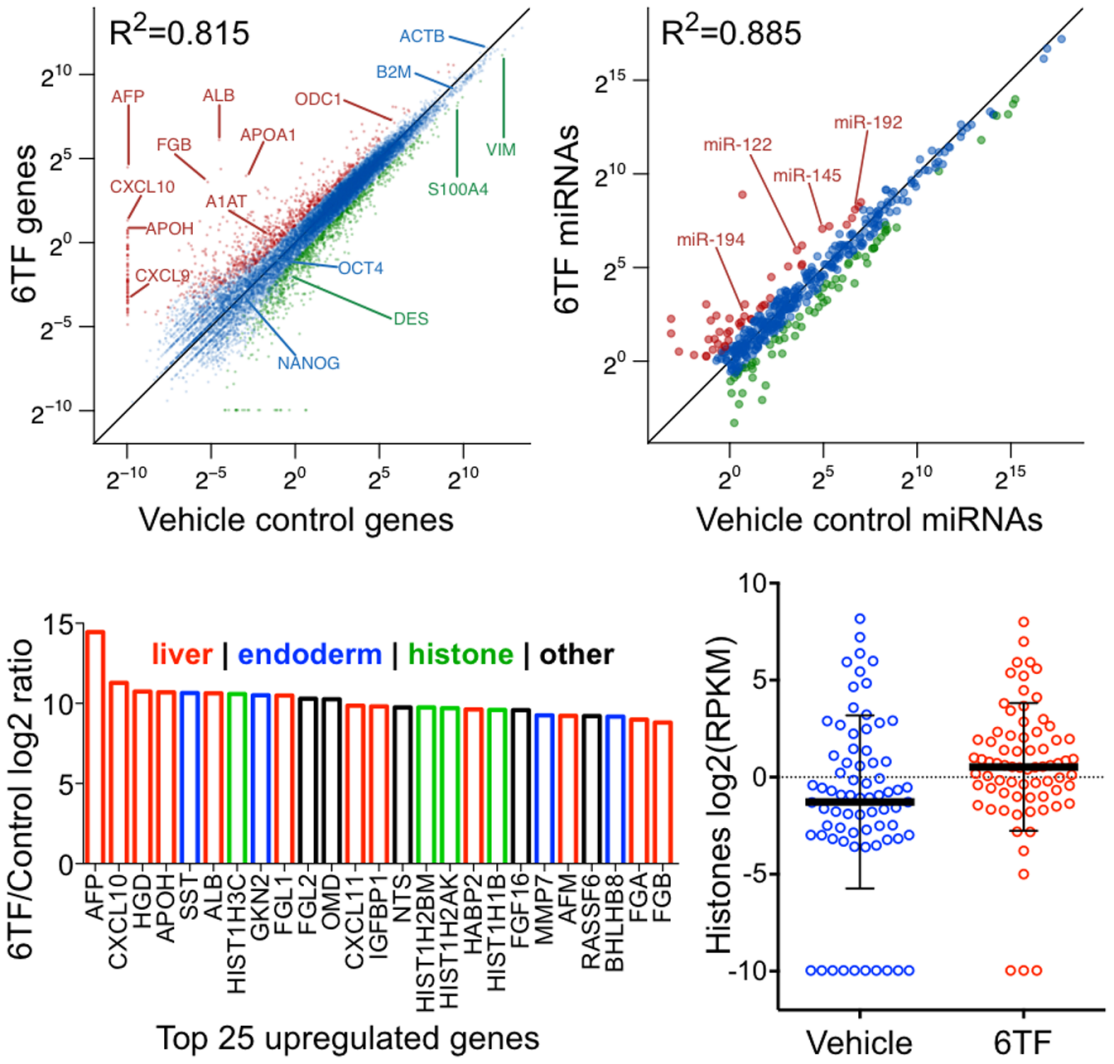
Global gene and small RNA sequencing analysis of reprogrammed cells. (A–B) Genes and small RNAs significantly upregulated more than 2-fold over control (red), significantly downregulated more than 2-fold below control (green), and genes without significant changes of 2-fold or more (blue) are plotted logarithmically for 6TF versus vehicle control. Well-known liver and liver-repair associated genes are upregulated (labeled in red), whereas fibroblast associated genes are downregulated (labeled in green). Pluripotency genes and control genes are unchanged (labeled in blue). Well-known hepatic miRNAs, such as miR-122, are upregulated (labeled in red). False discovery rates less than 0.001 for genes and p-values less than 0.05 for small RNAs were deemed significant. (C) Of the top 25 most upregulated genes in reprogrammed cells, twelve (nearly half) are associated with liver or liver-repair (red), four are associated with other endodermal tissues (blue), and four are histones (green). Histone genes were also globally upregulated in reprogrammed cells (D); mean and 75% and 25% quantiles are indicated. The full expression data for all genes (Tables S4, S5, and S6) and small RNAs (Tables S7, S8, and S9) are provided in the supporting information.

**Figure 4:**
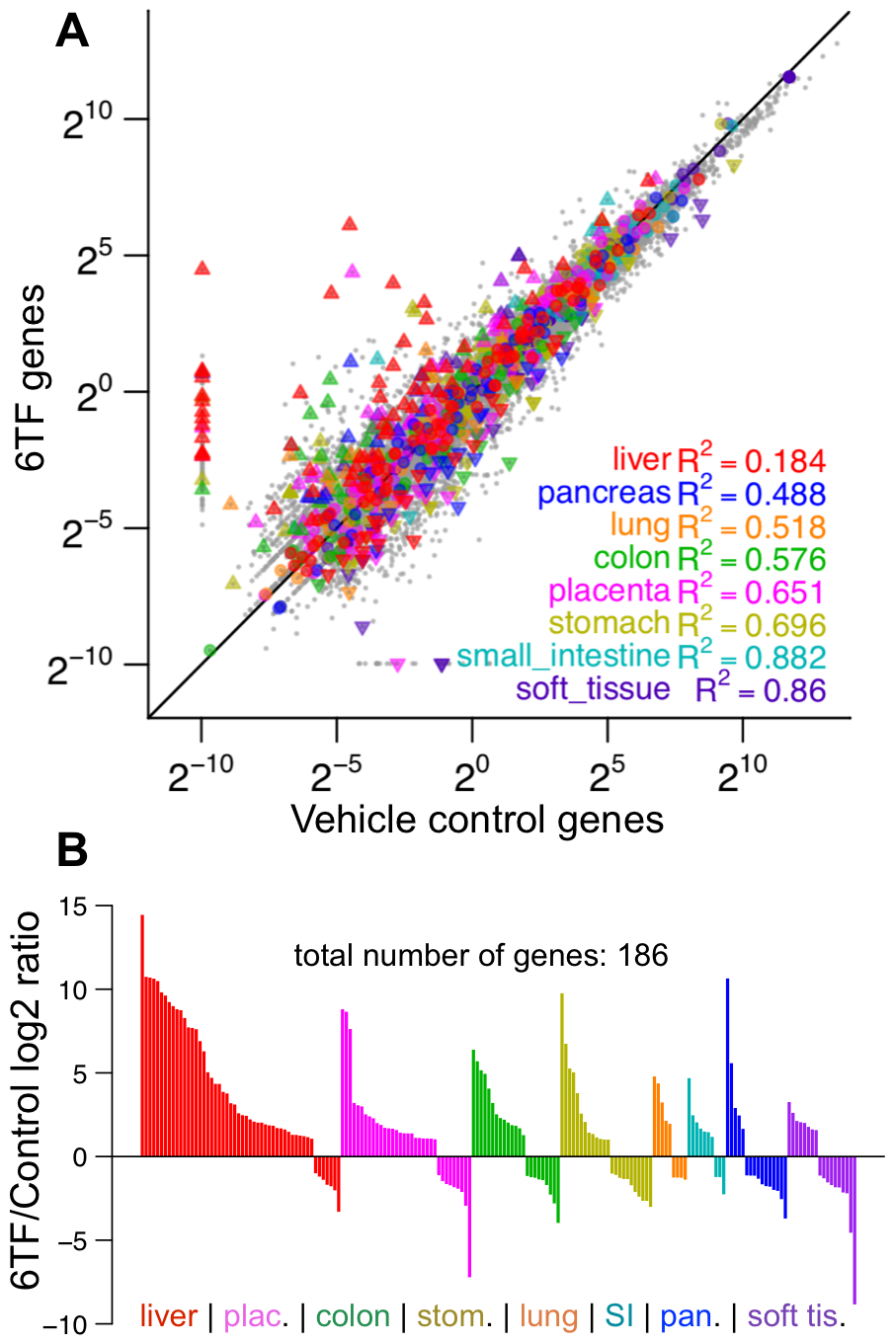
Liver-specific genes show more dispersion and upregulation than other endodermal tissue-specific genes. Genes annotated as specific to an endodermal tissue, placenta (proxy for cellular immaturity), or soft-tissue (proxy for fibroblasts) were taken from the TiGER database and plotted on a scatterplot (A), with up or down facing triangles indicating significant up or downregulation respectively. As indicated by the lower R^2^ value, liver-specific genes were highly dispersed away from control compared to other developmentally related endodermal tissues, such as pancreas. (B) Tissue-specific genes up or downregulated more than 2-fold were displayed as bars. Genes are grouped by tissue and ranked by expression level. Tissues were ordered based on the positive logarithmic area under the curve. The majority of dispersion of liver-specific genes resulted from upregulations. Other endodermal tissues were upregulated and downregulated proportionally. Soft tissue genes were primarily downregulated.

Global analysis of 6TF samples compared to vehicle control fibroblasts showed that hepatocyte-specific genes, such as *APOA1*, *APOH*, *FGB*, and *SERPINA1* (*A1AT*), were dramatically upregulated, while fibroblast-specific genes, such as *FSP1*, *DES*, and *VIM* were downregulated (Figure 3A). Notably, pluripotency genes, such as *OCT4* and *NANOG*, were unchanged. Genes involved in liver repair [24], [25], such as *CXCL9*, *CXCL10*, and *ODC1* were activated. Control genes, such as *ACTB* and *B2M*, were unchanged. Hepatocyte-associated miRNAs, such as miR-122, miR-145, miR-192, and miR-194, were also upregulated (Figure 3B). Notably, miR-122, which accounts for over 70% of the total miRNA of hepatocytes [26], was among the most upregulated miRNAs. Of the top 25 most upregulated genes in 6TF samples, twelve could be ascribed as liver-specific or liver repair-associated, whereas only four were associated with any other endodermal tissues (Figure 3C). Interestingly, four of the top 25 genes encoded histones, a trend also observed globally as genes encoding histones were expressed higher in 6TF cells than control (Figure 3D). To form an unbiased understanding of global changes in tissue-specific genes in early reprogramming, the Tissue-specific Gene Expression and Regulation (TiGER) database [27] was used to annotate genes as specific to a major endodermal tissue (colon, liver, lung, pancreas, small intestine, and stomach), placental tissue as proxy for cellular immaturity, and soft tissue as proxy for fibroblasts. In 6TF, genes that were annotated as liver-specific (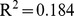) were most divergent from control, pancreas-specific genes a distant second (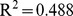), and soft tissue-specific genes nearly unchanged (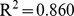) (Figure 4A). Examination of tissue-specific genes up or downregulated 2-fold or more revealed that dispersion of liver-specific genes resulted primarily from upregulation (Figure 4B). Whereas, dispersion of genes specific to other endodermal tissues followed no direction and soft tissue genes were primarily downregulated. Furthermore, many upregulated placenta-specific genes corresponded to fetal liver. Overall, sequencing demonstrated that reprogrammed cells preferentially moved toward a hepatic fate over other closely related endodermal fates and away from the starting soft tissue state of fibroblasts.

### *HNF1A* is necessary for reprogramming and sufficient in combination with two interchangeable factors

**Figure 5:**
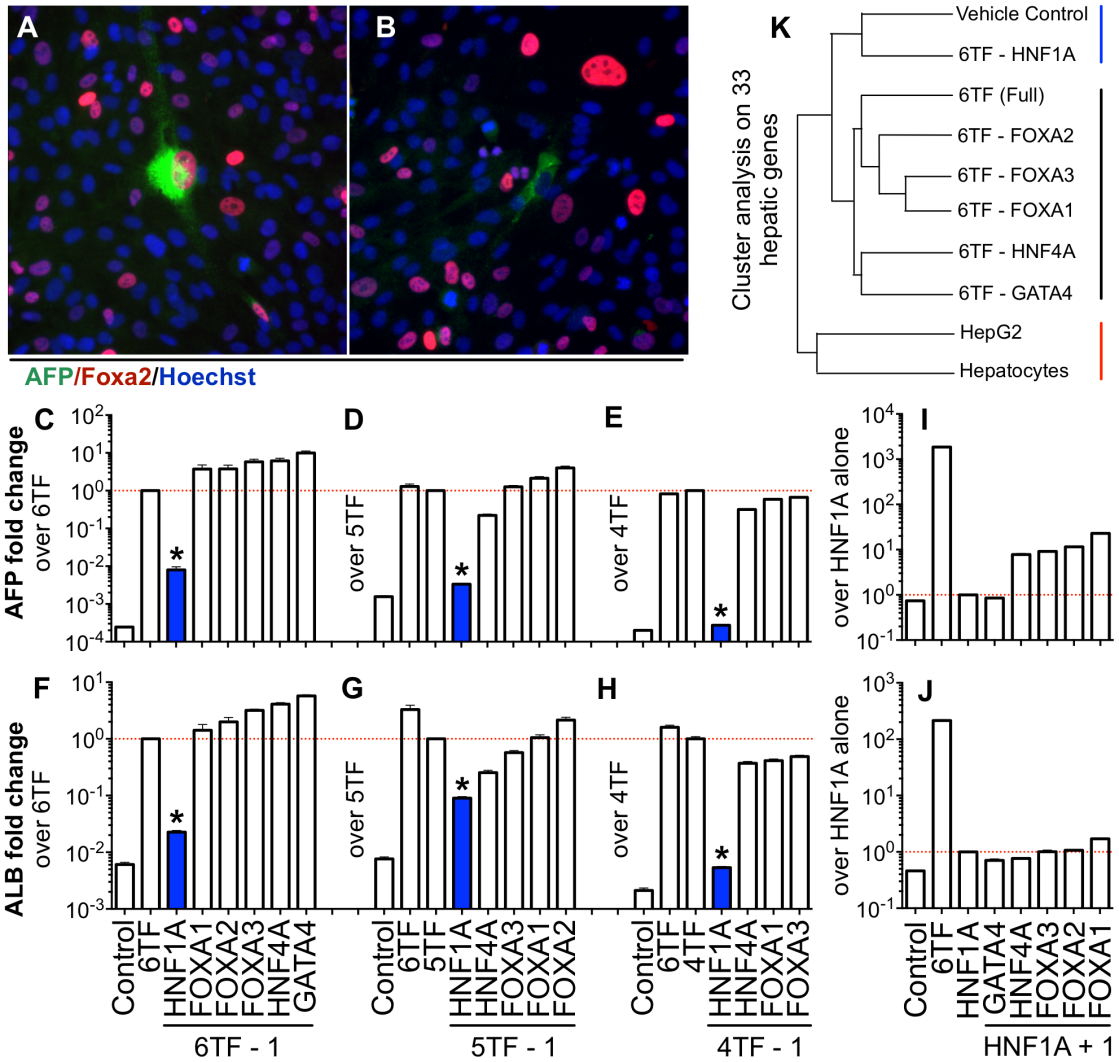
*HNF1A* is necessary for reprogramming and sufficient in combination with two interchangeable transcription factors. (A–B) Concurrent staining of reprogrammed samples for AFP and Foxa2 shows that AFP-positive converted cells can exist with or without Foxa2 receipt and expression, indicating that not all six factors of 6TF are necessary for reprogramming. Nuclei are stained blue by Hoechst. (C–E) Consistent loss of *AFP* induction with removal of *HNF1A* (blue) from reprogramming cocktails, 6TF, 5TF, and 4TF, with little or no decrease of induction due to removal of other factors. (F–H) Albumin induction measurement shows the same trend as *AFP* induction. Reprogramming can be achieved with as little as three transcription factors, *HNF1A* plus two of the following three factors, *FOXA1*, *FOXA3*, or *HNF4A*. Reprogramming could not be achieved at levels resemebling those of 6TF with less than three factors (I–J). Data points shown are mean
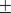
SD. Stars indicate p-val 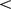
0.001. (K) 5TF sample lacking *HNF1A* clusters with vehicle control separately from 6TF and other 5TF samples and away from hepatocytes based on 33 hepatic genes, supporting the absolute necessity of *HNF1A*.

We next sought to understand which factors of 6TF were necessary for reprogramming. Concurrent staining of AFP and transfected factors after reprogramming confirmed that not all factors were required for conversion. While most AFP positive cells also stained for any given factor of 6TF (Figure 5A and S6), examples of cells that had not received all six factors yet still expressed AFP abounded (Figure 5B). Successive reductive experiments were performed, where one factor at a time was removed from the reprogramming cocktail and changes in *ALB* and *AFP* expression were measured. All experiments showed that *HNF1A* was consistently critical for reprogramming and that efficiency of conversion could be maintained with as little as three factors (Figure 5C–H). Possible combinations that maintained efficiency were *HNF1A* plus any two of the following three factors, *FOXA1*, *FOXA3*, or *HNF4A* (Figure 5E and 5H). An additive experiment, where each individual factor of 6TF was added back to *HNF1A* alone, showed that no combination of two factors could achieve significant conversion (Figure 5I–J). Cluster analysis of primary human hepatocytes, HepG2 cells, 6TF reprogrammed cells, vehicle control cells, and cells reprogrammed with all combinations of 6TF minus one factor (5TF) on 33 hepatic genes confirmed that *HNF1A* was essential to reprogramming (Figure 5K). The 5TF combination lacking *HNF1A* clustered with vehicle control, while all other 5TF combinations clustered with 6TF and closer to hepatocytes and HepG2 cells.

**Figure 6:**
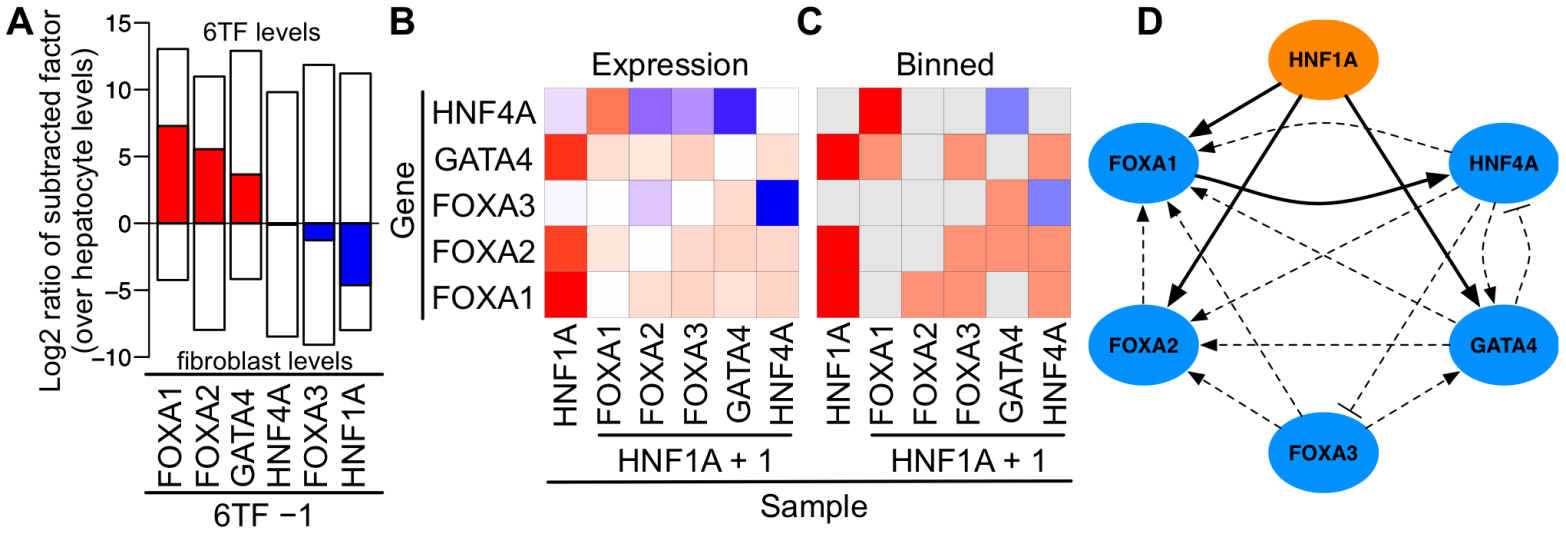
Robust compensation and interaction between the transcription factors of 6TF. (A) Cells were reprogrammed with six different combinations of 5TF. The factor missing from each 5TF sample is indicated on the x-axis, and its expression compared to primary hepatocytes is indicated (log2 ratio). The top of each clear bar indicates the level of each factor when delivered to fibroblasts via 6TF; the bottom of each clear bar indicates the endogenous level found in untransfected fibroblasts. Taken together these bars show the compensation each factor receives from the other five factors of 6TF, in relation to hepatocytes, fibroblasts, and 6TF samples. *FOXA1*, *FOXA2*, *GATA4*, and *HNF4A* were compensated at or above the levels found endogenously in hepatocytes. *FOXA3* levels were compensated near those of hepatocytes. However, *HNF1A* received almost no compensation with levels remaining closest to those found in fibroblasts. (B) The first column indicates the effects reprogramming with *HNF1A* alone exerts on the remaining five factors of 6TF over fibroblast control levels. The remaining columns indicate the effect reprogramming with *HNF1A* plus one additional factor have on the remaining four factors over the levels found when cells are reprogrammed with *HNF1A* alone. Upregulations are in red and downregulations in blue. Extensive interactions between the factors are observed. (C) The interactions of the previous matrix are binned into bright red activations (10-fold or more increase in expression), light red prospective activations (2-fold or more increase), and light blue prospective inhibitions (2-fold or more decrease). (D) The interactions defined by the previous binned matrix represented as a diagram between the factors of 6TF during reprogramming. Prospective interactions are dashed.

We hypothesized that differences in necessity among the factors were driven by cross-activation and compensation. In particular, we expected *HNF1A* to be the least compensated factor of 6TF. Removal of a factor from 6TF and subsequent measure of its gene expression during reprogramming served to quantify compensation and revealed that while all other factors were compensated above or near the levels normally found in a hepatocyte, *HNF1A* levels remained closer to those of a fibroblast (Figure 6A). In order to understand the specific interactions guiding compensation, we constructed a matrix of conditions to characterize how *HNF1A* alone or *HNF1A* plus an additional factor influenced the expression of the remaining factors over vehicle control or *HNF1A* alone respectively (Figure 6B). We binned upregulations of 10-fold or more as activations and up or downregulations of less than 10-fold but greater than 2-fold as prospective activations or inhibitions respectively (Figure 6C). We identified four activations, ten prospective activations, and two prospective inhibitions. Based on these interactions, we constructed a network diagram that revealed robust cross-activation and redundancy between the six factors (Figure 6D), supporting our hypothesis that necessity and sufficiency of the factors are guided by cross-activation and compensation.

## Discussion

We have demonstrated that a combination of *HNF1A* along with two additional interchangeable factors is sufficient to reprogram human fibroblasts specifically to a hepatocyte-like state. This is notably different from the minimal cocktails determined by those previously characterizing hepatic reprogramming in the mouse [9], [10]. Thus as reported for neural conversion [28], the transcriptional circuitry guiding hepatic conversion in human is different from that of the mouse. Moreover, while our work was under review, two groups independently published on reprogramming human fibroblasts to hepatocytes using lentiviruses to deliver the desired factors [29], [30]. Each group concludes slightly different final cocktails of reprogramming factors are necessary and sufficient. Huang et al. settle on *HNF1A*, *FOXA3*, and *HNF4A*. This is among the possible combinations we reach of *HNF1A* plus two of the factors *FOXA3*, *HNF4A*, and *FOXA1*. While Du et al. reach a slightly more distinct set from Huang et al. and our findings, notably, *HNF1A* is still among the factors determined to be necessary and sufficient. Therefore, the studies by Huang et al. and Du et al. confirm our findings, particularly the importance of *HNF1A*. In our analysis, we characterize why *HNF1A* is so crucial for reprogramming by dissecting the transcriptional interactions guiding the observed necessities and sufficiencies. Specifically, we show that *HNF1A* strongly activates *FOXA1*, *FOXA2*, and *GATA4* and that *FOXA1* strongly activates *HNF4A*. Thus, by including *HNF1A*, *FOXA3*, and *FOXA1* or *HNF4A* (three factors total), one can either force-overexpress or strongly activate all six factors of 6TF with only three factors. This helps to rationally explain our findings that a combination of *HNF1A* plus two additional factors is sufficient for hepatic reprogramming in human.

The methods we have described here have numerous advantages over traditional methods relying on retroviral vectors and are of great potential for the therapeutic application of the direct reprogramming field. We would therefore like to note that working extensively with RNA and handling cells in antibiotic-free media (during daily RNA transfection) are technically challenging methods. These methods are highly sensitive to small changes and perturbations that may seem insignificant. For this reason, we recommend that all attempts to replicate these methods strictly follow our protocols without deviation until results have been successfully replicated. Particularly, all DNA templates constructed for IVT should be sequence confirmed and band-purified. Also, mmRNAs should be thoroughly quality checked as per the guidelines described in our methods. Finally, we would like to note that efficiency is dependent upon how strictly daily transfection is followed. Transfections should be performed at the same time each day. As mRNA delivery is the limiting step in terms of reprogramming efficiency, synthetic mRNA reprogramming has the potential to increase dramatically in efficiency with improvements in transfection methods and reagents. In particular, a reagent that couples multiple mRNAs to one particle, such as bead or nanoparticle, would have major implications for reprogramming efficiency. An increase in transfection efficiency of one factor results in an exponential increase in reprogramming efficiency, since reprogramming depends on concerted receipt of multiple factors by each cell.

We term our reprogrammed cells, hepatocyte-like cells, as neither a fully immature or fully mature state can be ascribed. Hepatocyte-like cells express markers of immaturity, such as *AFP*, as well as many marker of maturity, such as *ALB*, *APOA1*, and *SERPINA1*. Their global RNA expression profile suggests they are closer to mature liver than fetal liver but that nevertheless a clear fetal liver program is also active. Future studies will surely be required to characterize the maturity and function of our hepatocyte-like cells metabolically and *in vivo*. However, our results provide a mechanistic analysis of the interactions between the factors necessary and sufficient to produce a hepatocyte-like state. Additionally, by unbiased global analysis, we demonstrate that reprogramming with our factors produces a truly hepatocyte specific conversion. Finally, to the best of our knowledge, we demonstrate the first direct reprogramming of any kind without genomic alteration or risk of alteration by using synthetic modified mRNAs to overexpress reprogramming factors, a technique that can also be extended to other non-hepatic conversions. These advances not only bring induced hepatocytes closer to a therapeutic reality for the treatment of human liver diseases, but also provide a platform from which the entire field of direct reprogramming can overcome its retroviral reliance and be translated to its ultimate applications in regenerative medicine.

## Materials and Methods

### Cell Culture

CD34+ bone marrow cells (AllCells, Emeryville, CA) were received fresh and were plated and expanded immediately upon receipt. Cells were expanded for 3–5 days in StemSpan SFEM (StemCell Technologies, Vancouver, Canada) before being passaged for media formulation experiments. StemSpan SFEM media was supplemented with 100 ng/mL SCF, 100 ng/mL FLT3, 25 ng/mL IL6, and 25 ng/mL IL3 (Peprotech, Rocky Hill, NJ) during expansion. Human embryonic stem cell line SA181 (Cellartis AB, Goteborg, Sweden) was cultured on matrigel (Corning, Tewksbury, MA) coated flasks in TesR2 medium (StemCell Technologies, Vancouver, BC) during expansion. ESCs were split for reprogramming experiments using TrypLE (Life Technologies, Grand Island, NY). Human adult female cryopreserved hepatocytes (Product No. F00995, Celsis/In Vitro Technologies, Chicago, IL) were thawed in CHRM medium (Invitrogen, Carlsbad, CA) and plated in Williams medium E supplemented with Primary Hepatocyte Thawing and Plating Supplement Pack (Invitrogen). BJ fibroblasts (Stemgent, Cambridge, MA) and MRC-5 fibroblasts (ATCC, Manassas, VA) were thawed and plated directly into DMEM/F12+Glutamax (Invitrogen) with 10% HyClone FBS (Thermo Scientific, Waltham, MA), 1% Insulin-Transferrin-Selenium (Invitrogen), 1% MEM Non-Essential Amino Acids (Invitrogen), and 5 mM HEPES buffer. After a two day expansion, cells were dissociated with 0.5% Trypsin-EDTA (Invitrogen) and plated at 1,000 cells/cm2 on Collagen-I coated plates (BD Biosciences, San Jose, CA) for reprogramming experiments. For hepatic reprogramming experiments, media was supplemented with 20 ng/mL human hepatocyte growth factor (HGF), 20 ng/mL epidermal growth factor (EGF), 20 ng/mL fibroblast growth factor 2 (FGF2) (Peprotech), 200 ng/mL B18R (eBioscience, San Diego, CA), and 0.1 uM dexamethasone (Sigma, St. Louis, MO). For experiments comparing the effects of dexamethasone on reprogramming efficiency, dexamethasone, where used, was supplemented at 0.1 uM for regular concentrations and 1 uM for high concentrations. For *TLR3* activation experiments, PolyI:C (Tocris, Bristol, UK) was supplemented upon transfection at 300 ng/mL. Upon reaching confluence during reprogramming experiments, wells were sub-cultured at a 1:6 ratio. All cell culture was performed in antibiotic-free media.

### DNA Template Construction for *in vitro* Transcription (IVT)

The process of IVT template construction is diagramed in Figure 1. All primers and splints used for PCRs and ligations are listed in Table S2. Synthetic alpha-globin 5 and 3UTR sequences used were as previously described [18]. All oligos, including primers, splints, and UTRs were synthesized in house at the Genentech oligo synthesis core facility. Open reading frame (ORF) PCR amplifications of DNA encoding C/EBPA FOXA1, FOXA2, FOXA3, GATA4, GATA6, HHEX, HNF1A, HNF1B, HNF4A, and HNF6Awere templated from DNA plasmids containing each of the respective human ORFs (Origene, Rockville, MD). ORF PCR for nuclear localization sequence GFP (NLS-GFP) was templated from pturboGFP plasmid (Evrogen through Axxora, Richmond, VA). The nuclear localization sequence for NLS-GFP was added to the N-terminal end using a modified forward primer. ORF PCRs and ligations were performed as previously described [18]. To maximize ligation efficiency, forward ORF primers were 3 phosphorylated, and the 3UTR was 5 phosphorylated upon oligo synthesis. Intermediate ORF PCR and ligation products were purified using QIAquick PCR purification columns (Qiagen, Valencia, CA). Products of successful ligations were simultaneously selected for, amplified, and polyA-tailed by template-based forward and reverse tailing primers. Final template PCR products were run out on 1.2% Agarose SYBR E-Gels (Invitrogen). Bands of the correct length were excised and purified sequentially using QIAquick Gel Extraction and QIAquick PCR purification columns (Qiagen). Fully purified templates were then confirmed to be error-free by DNA sequencing in the Genentech sequencing core facility. Fully purified, length and sequence-validated, DNA templates were then used for modified mRNA synthesis. A detailed stepwise protocol is available at http://dx.doi.org/10.6084/m9.figshare.1040039.

### Modified mRNA Synthesis

RNA was synthesized using the MEGAscript T7 kit (Ambion, Austin, TX), with 1.5 ug DNA template per each 40 uL reaction. IVT reactions were incubated either for 14–16 hrs at 30C or 3–6 hrs at 37C and DNased as described by the manufacturer. A modified ribonucleoside blend was used during IVT reactions, and RNA was subsequently purified, phosphatased, and re-purified as previously described [18]. RNA length and purity was assessed using an RNA 6000 Pico Kit with an Agilent 2100 Bioanalyzer (Agilent Technologies, Santa Clara, CA) and 1% Agarose SYBR E-Gels EX (Invitrogen). RNA concentration was determined by Nanodrop (Thermo Scientific) and adjusted to a stock concentration of 200 ng/uL by addition of Nuclease-free water (Ambion). A GFP encoding mRNA from Maxcyte was used for non-nuclear GFP transfection (Gaithersburg, MD). A detailed stepwise protocol is available at http://dx.doi.org/10.6084/m9.figshare.1040040.

### Modified mRNA Transfection

TransIT-mRNA (Mirus Bio, Madison, WI) cationic lipid reagent was used for transfection. Before transfection, RNA was diluted 20-fold in Opti-MEM Reduced Serum Media (Invitrogen), and BOOST reagent was added at 2 uL per microgram of RNA, after which, TransIT-mRNA reagent was added at 2 uL per microgram of RNA. These RNA-lipid complexes were incubated at RT for 3 min and delivered to cells. Cell culture media was always changed immediately prior to transfection.

### Immunostaining

Cells were fixed in 4% formaldehyde for 15 min and washed 3 times for 5 min with PBS. Cells were blocked for 1 hr at RT in 5% Goat (Cell Signaling, Dansvers, MA) or Donkey (Sigma) Serum and 0.3% Triton X-100 (Sigma). Cells were stained for 2 hrs (primary antibodies) or 1 hr (secondary antibodies) at RT in 1% BSA (Sigma) and 0.3% Triton X-100. Cells were washed 3 times for 5 min with PBS after primary antibody incubation. FOXA1 (Abcam, Cambridge, MA), FOXA2 (Cell Signaling), and FOXA3 (Santa Cruz Biotech, Santa Cruz, CA) primary antibodies were used at 1∶50 dilutions; HNF4A (Cell Signaling), HNF1A (BD Biosciences), and albumin (Abnova, Walnut, CA) primary antibodies were used at 1∶100 dilutions; GATA4 (BD Biosciences) and alpha-fetoprotein (AFP) (Sigma) primary antibodies were used at 1∶200 dilutions. Anti-mouse, rabbit, and goat IgG Alexa Fluor 488 and 555 secondary antibodies (Invitrogen) were used at 1∶1000 dilutions. HCS LipidTOX Neutral Lipids Stain (Invitrogen) was used for lipid droplet staining as directed by the manufacturer. Hoechst 33342 (Invitrogen) was used at 1 ug/mL for all nuclear staining. Images were acquired with an IX81 Inverted microscope (Olympus, Center Valley, PA). For images comparing GFP and nuclear GFP expression across RNA transfection concentration, all images were captured using the same parameters and batch renormalized using SlideBook 5. For images from which no comparisons were drawn, small channel adjustments were made to the entire image in order to optimize intensity of the blue, green, and red channels.

### qPCR Gene Expression

For reductive and additive experiments, RNA was isolated directly from cell culture wells using the miRNeasy Mini kit (Qiagen). 200 ug RNA from each sample was used in 50 uL RT reactions from the Cells-to-Ct kit (Ambion). For all other experiments, the Cells-to-Ct kit was used for RNA extraction, and 22.5 uL of this was carried over to 50 uL RT reactions. Before RT, RNA samples were DNased according to the respective manufacturer instructions. For qPCR, 4 uL of each RT was used in 20 uL reactions with Taqman Universal Master Mix, no UNG (Applied Biosystems, Foster City, CA). Primer/probes used were 20x Taqman Gene Expression Assays (Applied Biosystems) and are listed in Table S3. Where two different assays were used for the same gene, an average of both assays was taken. Either the ViiA 7 Real Time PCR System (Applied Biosystems) or the Biomark HD System (Fluidigm, South San Francisco, CA) was used to perform and analyze qPCRs. Experiments showing only *ALB* and *AFP* gene expression were run on the ViiA 7 Real Time PCR System, and *RPL19* gene expression was used as the endogenous control. For these experiments, vehicle control samples did not show any detectable *AFP*. For comparison purposes an artificial Ct of 40 was applied to these samples. Further downstream calculations were performed as standard. Factor compensation experiments and experiments involving larger panels of genes were performed on the Biomark HD System, and an average of *RPL19*, *B2M*, and *GAPDH* gene expression was used for endogenous control. Genes that were not detectable for a particular sample and for which an artificial Ct value was applied for comparison purposes were indicated in the appropriate Figure. For such cases, the artificial Ct applied was equal to the maximum Ct observed amongst all samples for the relevant gene plus 1. Artificial Ct values applied in both cases were conservative and corresponded to the maximum possible value of expression of the non-detected gene. In Figures 2 and S2, replicates are biological, along with 2 technical replicates for each biological replicate. In Figures 5, 6, S1, and S3, replicates are technical.

### Transcriptome and Small RNA Sequencing

Total RNA samples were extracted by miRNAeasy Mini Kit (Qiagen) and were provided to the Bejing Genomics Institute Americas (BGI Americas, Cambridge, MA) for transcriptome (4G clean data) and small RNA (20mil clean reads) sequencing. RNA samples were processed by the standard BGI workflow, including, RNA quality assessment, library construction, library validation, clustering, sequencing on Illumina HiSeqTM 2000, and standard bioinformatics analysis. Significance of differentially expressed genes was determined by BGI by calculating the false discovery rate (FDR) for each gene using Bonferroni correction of p-values. FDR values under 0.001 were deemed significant. Differentially expressed small RNAs with p-values under 0.05 and expression differences larger than 2-fold were deemed significant. All additional analyses and graphing of data were performed in R. Where log2 ratios are plotted, these correspond to the log2 of the appropriate RPKM values. Tissue specific genes were identified using the Tissue-specific Gene Expression and Regulation (TiGER) database [27].

### Reprogramming Experiments

For initial 11TF and 6TF induction experiments, a modified mRNA pool with a molar ratio of 1∶1 between each factor was used. A volumetric ratio of 74:113:59:107:91:[93:91:72:88:119:93] (*CEBPA*:*GATA6*:*HHEX*:*HNF1B*:*HNF6A*:[*FOXA1*:*FOXA2*:*FOXA3*:*GATA4*:*HNF1A*:*HNF4A*]) was used for pooling. A total amount of 900ng per well (6-well plate) was transfected daily at the same time each day for 5–9 days. For reductive and additive experiments, factors were subtracted from the 6TF pool as appropriate by keeping the final amount of each individual factor but not the final total RNA amount constant. All compensation, reductive, and additive experiments were performed by daily administration of the relevant reprogramming cocktail for 5–6 days.

## Supporting Information

Figure S1
**Proper receipt, translation, and localization of reprogramming transcription factors delivered as synthetic mmRNAs.** (A) High-levels of reprogramming factors were maintained with daily transfection 6TF mmRNAs for 9 days (mean
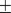
SD). (B–G) Translation and proper localization of factors after 6TF mmRNA transfection.(TIFF)

Figure S2 **Hepatocyte-like cells with proper localization of albumin.** The vehicle column indicates BJ fibroblasts receiving vehicle control daily for 5 days. The 6TF column indicates BJ fibroblasts receiving 6TF mmRNAs daily for 5 days. Hoechst localizes properly to the nucleus. Distinct albumin-positive hepatocyte-like cells appear only in the 6TF transfected fibroblast samples. Albumin localizes correctly to the cytoplasm, remaining outside of the nucleus.
(TIFF)

Figure S3 **Hepatocyte-like cells with proper localization of AFP.** The vehicle column indicates BJ fibroblasts receiving vehicle control daily for 5 days. The 6TF column indicates BJ fibroblasts receiving 6TF mmRNAs daily for 5 days. Hoechst localizes properly to the nucleus. Distinct AFP-positive hepatocyte-like cells appear only in the 6TF transfected fibroblast samples. AFP localizes correctly to the cytoplasm, remaining outside of the nucleus. Cells appear healthy in the phase image. The AFP-positive hepatocyte-like cells display some morphological differences from the surrounding fibroblasts, such as less elongation.
(TIFF)

Figure S4
**Appearance of binucleated cells and hepatic gene expression in reprogrammed neonatal and fetal fibroblasts and embryonic stem cells.** (A) Binucleated cells were observed at a frequency of around 1∶10,000 in 6TF reprogrammed wells but nearly unobserved in vehicle control wells. (B) qPCR gene expression of 33 hepatic genes in primary adult hepatocytes and 6TF reprogrammed neonatal fibroblasts (BJ) over vehicle control (mean
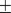
SD). (C) The same expression analysis performed on fetal fibroblasts (MRC5). For both reprogrammed neonatal and fetal fibroblasts, expression levels were generally between those of control fibroblasts and primary hepatocytes. (D) Induction of AFP and albumin in ESCs after 5 days of reprogramming with 6TF (mean
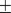
SD).(TIFF)

Figure S5
**Effects of reprogramming media and TLR3 induction on reprogramming.** (A–B) Fibroblasts grown in reprogramming media without concurrent 6TF transfection did not have increases in ALB or AFP compared with those grown in basal media alone (unsupplemented with growth factors and dexamethasone). However, reprogramming media displayed a striking synergy when 6TF was transfected daily. Reprogramming media along with 6TF transfection produced increases many 100-fold (ALB) and 10,000-fold (AFP) greater than the 6TF transfection scheme in basal media. (A) Removal of dexamethasone from reprogramming media caused an approximately 100-fold decrease in albumin induction for 6TF reprogrammed cells and a smaller but noticeable decrease for 11TF reprogrammed cells (mean
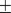
SD). Further supplementation with dexamethasone did not improve efficiency. (D) mmRNAs are sufficient to activate TLR3 higher than positive inducer, poly I:C. Further activation of TLR3 using poly I:C in addition to mmRNA transfection does increase TLR3 activation but does not improve albumin or AFP induction and is hence unnecessary (mean
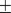
SD).(TIFF)

Figure S6 **Expanded image of** **Figure 5A** **shows multiple healthy hepatocyte-like cells.** Image (10× magnification) was taken in the same area as the image in Figure 5A (20× magnification). Hepatocyte-like cells arise during reprogramming primarily as singular events, surrounded by large unconverted fibroblast populations. The cells appear robust in the phase image.
(TIFF)

Table S1 **Hepatic reprogramming media composition.**
(PDF)

Table S2 **Oligos used for construction of DNA templates for** ***in vitro*** **transcription.**
(PDF)

Table S3 **List of Taqman assays used for qPCR gene expression.**
(PDF)

Table S4 **Complete set of genes detectable in BJ fibroblasts after reprogramming for five days (6TF versus vehicle control).**
(CSV)

Table S5 **Complete set of genes detectable in BJ fibroblasts after reprogramming for five days (11TF versus vehicle control).**
(CSV)

Table S6 **Complete set of genes detectable in BJ fibroblasts after reprogramming for five days (6TF versus 11TF).**
(CSV)

Table S7 **Complete set of small RNAs detectable in BJ fibroblasts after reprogramming for five days (6TF versus vehicle control).**
(CSV)

Table S8 **Complete set of small RNAs detectable in BJ fibroblasts after reprogramming for five days (11TF versus vehicle control).**
(CSV)

Table S9 **Complete set of small RNAs detectable in BJ fibroblasts after reprogramming for five days (6TF versus 11TF).**
(CSV)
