# Supplementary figures and images for: Direct Reprogramming of Human Fibroblasts to Hepatocyte-Like Cells by Synthetic Modified mRNAs
Source: PLoS One. 2014 Jun 25;9(6):e100134. doi: 10.1371/journal.pone.0100134 (PMC4070971; doi:10.1371/journal.pone.0100134)

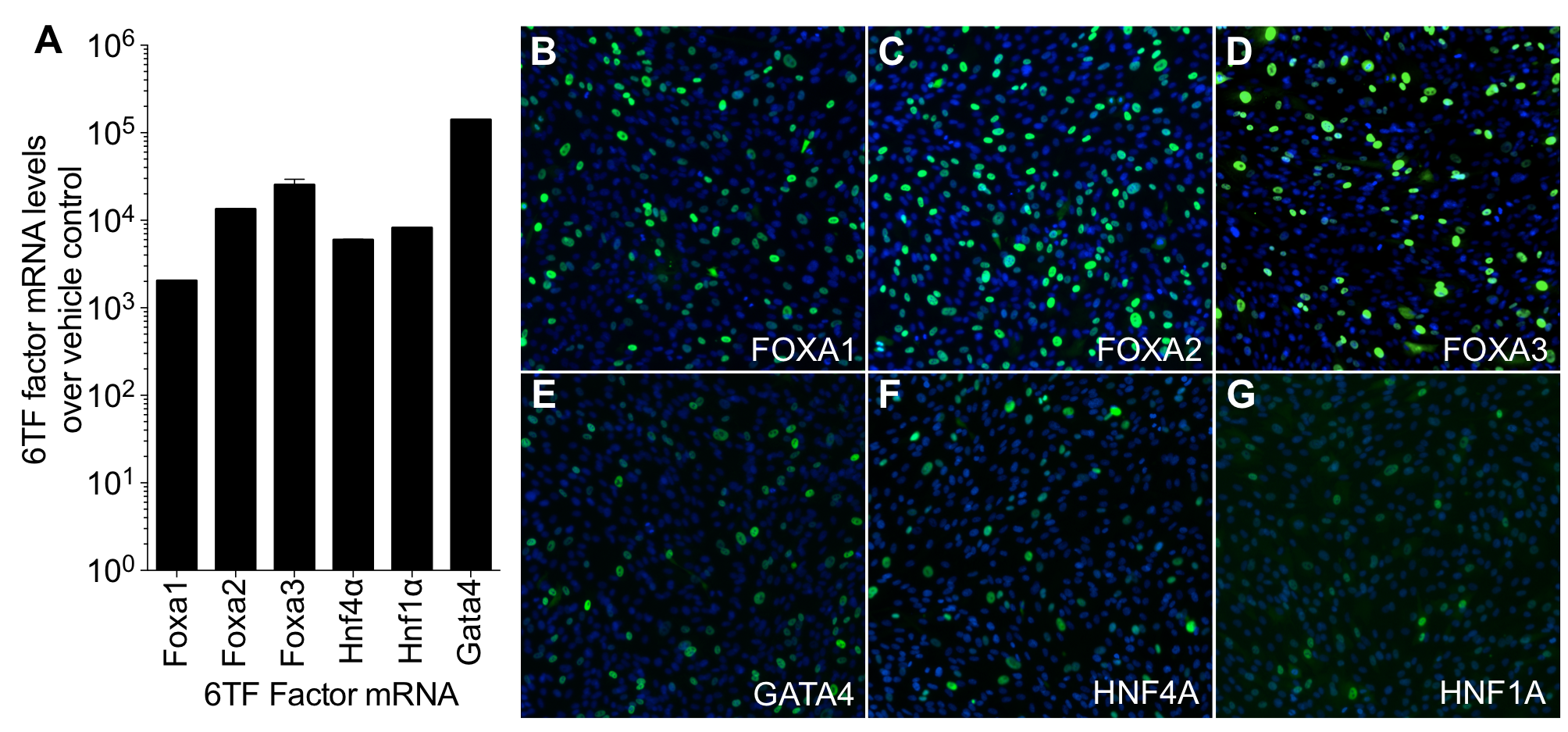

Supplement: Figure S1 — Proper receipt, translation, and localization of reprogramming transcription factors delivered as synthetic mmRNAs. (A) High-levels of reprogramming factors were maintained with daily transfection 6TF mmRNAs for 9 days (meanSD). (B–G) Translation and proper localization of factors after 6TF mmRNA transfection. (TIFF) [file pone.0100134.s001.tiff]

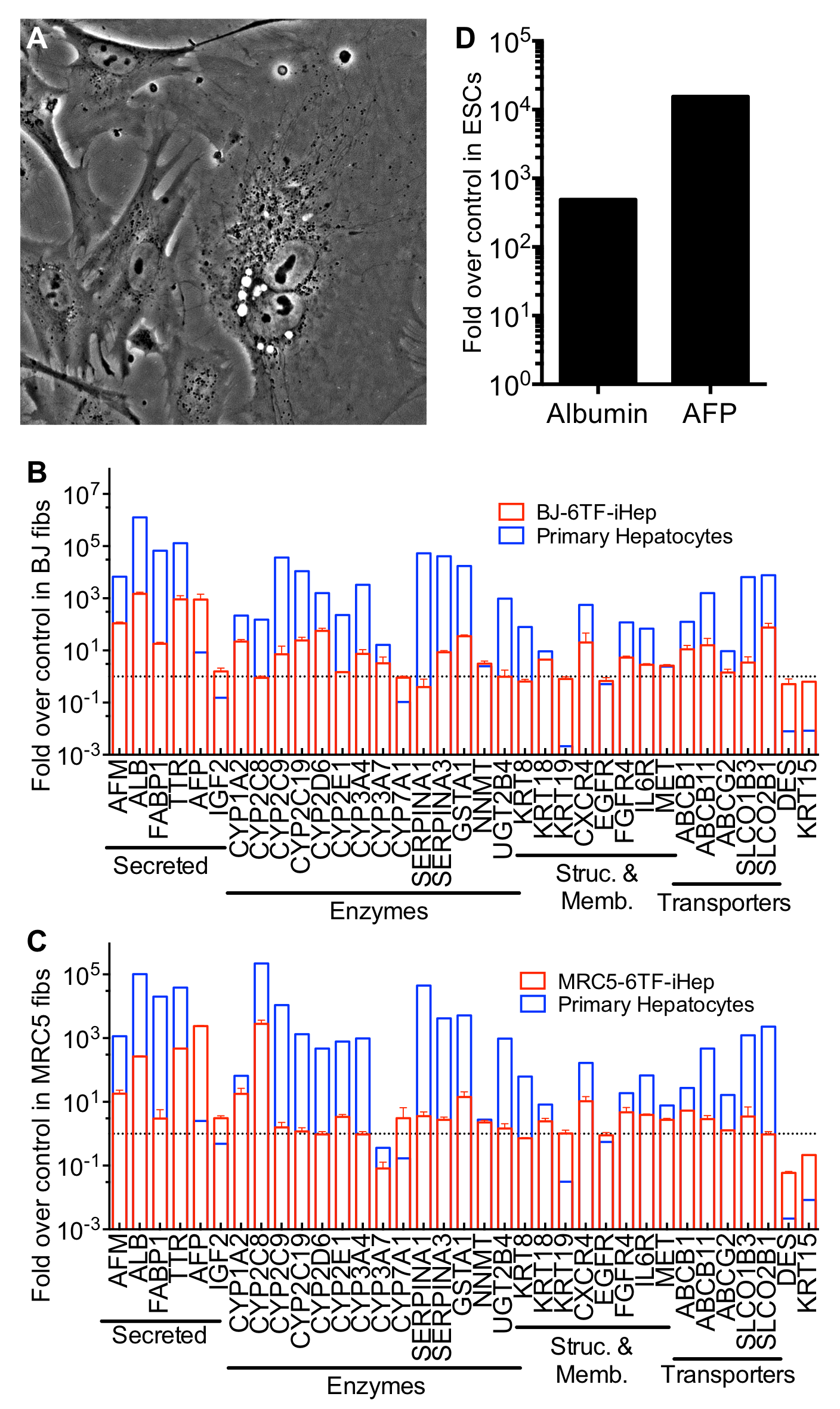

Supplement: Figure S4 — Appearance of binucleated cells and hepatic gene expression in reprogrammed neonatal and fetal fibroblasts and embryonic stem cells. (A) Binucleated cells were observed at a frequency of around 1∶10,000 in 6TF reprogrammed wells but nearly unobserved in vehicle control wells. (B) qPCR gene expression of 33 hepatic genes in primary adult hepatocytes and 6TF reprogrammed neonatal fibroblasts (BJ) over vehicle control (meanSD). (C) The same expression analysis performed on fetal fibroblasts (MRC5). For both reprogrammed neonatal and fetal fibroblasts, expression levels were generally between those of control fibroblasts and primary hepatocytes. (D) Induction of AFP and albumin in ESCs after 5 days of reprogramming with 6TF (meanSD). (TIFF) [file pone.0100134.s004.tiff]

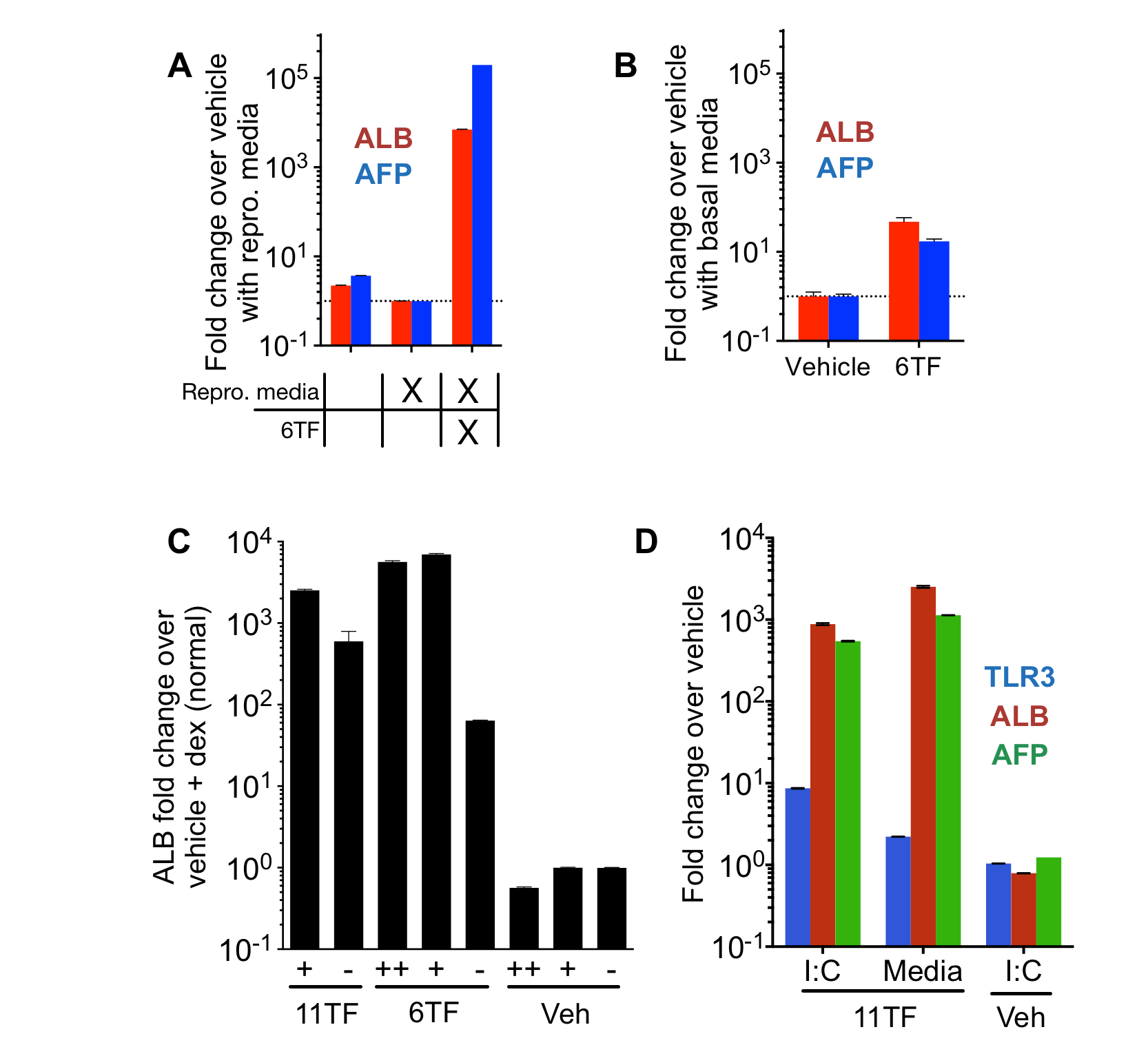

Supplement: Figure S5 — Effects of reprogramming media and TLR3 induction on reprogramming. (A–B) Fibroblasts grown in reprogramming media without concurrent 6TF transfection did not have increases in ALB or AFP compared with those grown in basal media alone (unsupplemented with growth factors and dexamethasone). However, reprogramming media displayed a striking synergy when 6TF was transfected daily. Reprogramming media along with 6TF transfection produced increases many 100-fold (ALB) and 10,000-fold (AFP) greater than the 6TF transfection scheme in basal media. (A) Removal of dexamethasone from reprogramming media caused an approximately 100-fold decrease in albumin induction for 6TF reprogrammed cells and a smaller but noticeable decrease for 11TF reprogrammed cells (meanSD). Further supplementation with dexamethasone did not improve efficiency. (D) mmRNAs are sufficient to activate TLR3 higher than positive inducer, poly I:C. Further activation of TLR3 using poly I:C in addition to mmRNA transfection does increase TLR3 activation but does not improve albumin or AFP induction and is hence unnecessary (meanSD). (TIFF) [file pone.0100134.s005.tiff]

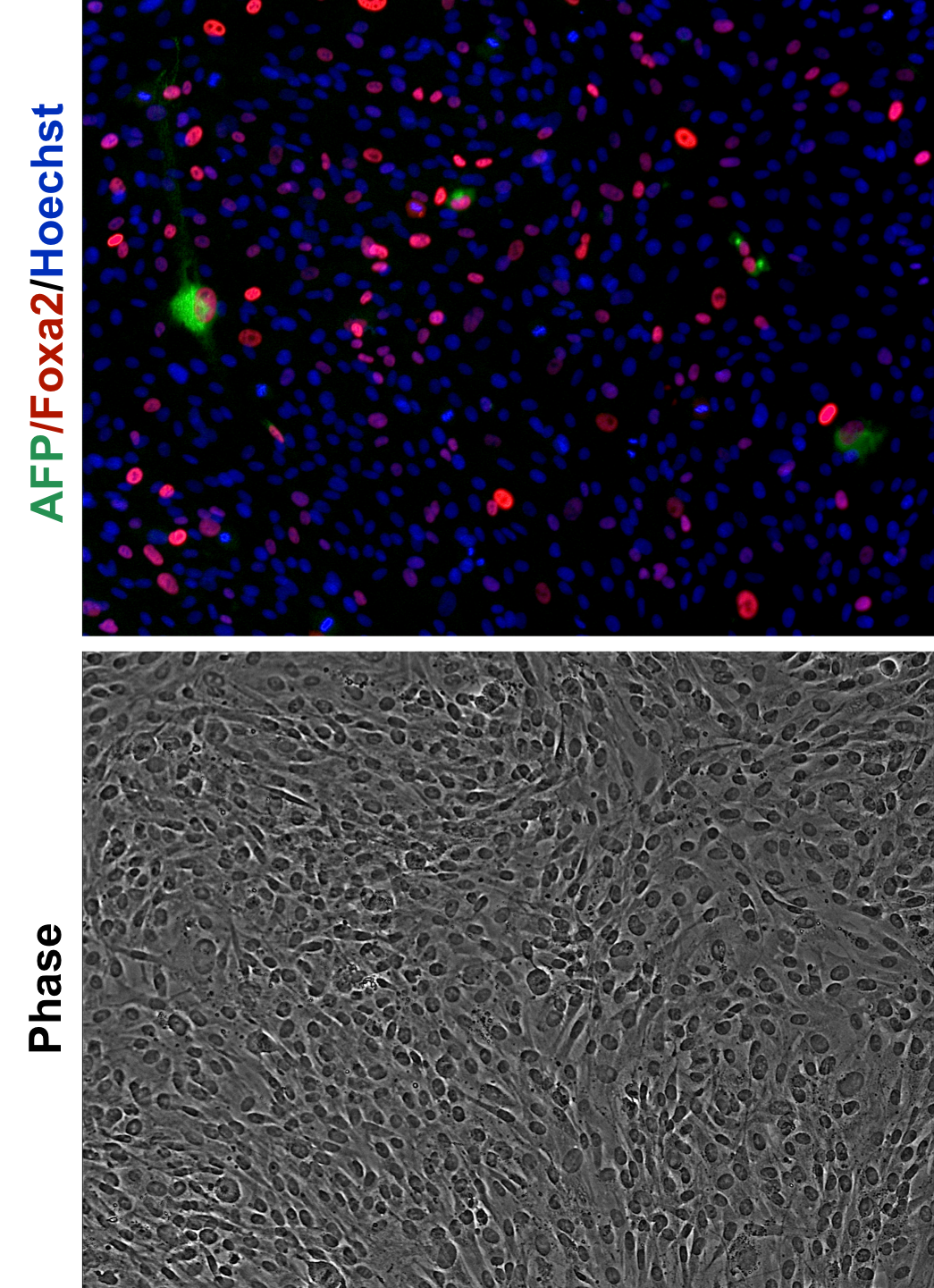

Supplement: Figure S6 — Expanded image of Figure 5A shows multiple healthy hepatocyte-like cells. Image (10× magnification) was taken in the same area as the image in Figure 5A (20× magnification). Hepatocyte-like cells arise during reprogramming primarily as singular events, surrounded by large unconverted fibroblast populations. The cells appear robust in the phase image. (TIFF) [file pone.0100134.s006.tiff]
