# Supplementary material for: Direct Reprogramming of Human Fibroblasts to Hepatocyte-Like Cells by Synthetic Modified mRNAs
Source: PLoS One. 2014 Jun 25;9(6):e100134. doi: 10.1371/journal.pone.0100134 (PMC4070971; doi:10.1371/journal.pone.0100134)
Supplement: Table S1 — Hepatic reprogramming media composition. (PDF) [file pone.0100134.s007.pdf]

Table S1. Hepatic reprogramming media and environment.

DMEM/F12 + Glutamax	
Supplements	
FBS	10%
ITS	1%
NEAA	1%
HEPES	5mM
Cytokines	
HGF	20ng/mL
EGF	20ng/mL
FGF2	20ng/mL
B18R	200ng/mL
Other	
Dexamethasone	0.1uM
Collagen-I coated plates	
Antibiotic free	
