# Supplementary material for: Direct Reprogramming of Human Fibroblasts to Hepatocyte-Like Cells by Synthetic Modified mRNAs
Source: PLoS One. 2014 Jun 25;9(6):e100134. doi: 10.1371/journal.pone.0100134 (PMC4070971; doi:10.1371/journal.pone.0100134)
Supplement: Table S2 — Oligos used for construction of DNA templates for in vitro transcription. (PDF) [file pone.0100134.s008.pdf]

Table S2. Oligos used for IVT

	ORF Forward Primer	ORF Reverse Primer
CEBPA	GAGTCGGCCGACTTCTACG	TCACGCGCAGTTGCCCAT
FOXA1	TTAGGAACTGTGAAGATGGAAGG	CTAGGAAGTGTTTAGGACGGGTCT
FOXA2	CACTCGGCTTCCAGTATGCT	TTAAGAGGAGTTCATAATGGGC
FOXA3	CTGGGCTCAGTGAAGATGGA	CTAGGATGCATTAAGCAAAGAGC
GATA4	TATCAGAGCTTGGCCATGG	TTACGCAGTGATTATGTCCCC
GATA6	GCCTTGACTGACGGCGG	TCAGGCCAGGGCCAGGG
HHEX	CAGTACCCGCACCCCG	TCATCCAGCATTAAAATAGCTTTTATC
HNF1A	GTTTCTAAACTGAGCCAGCTGC	TTACTGGGAGGAAGAGGCC
HNF1B	GTGTCCAAGCTCACGTCG	CCAGGCTTGTAGAGGACACTG
HNF4A	CGACTCTCCAAAACCCTCGT	CTAGATAACTTCCTGCTTGGTGA
HNF6A	AACGCGCAGCTGACCAT	TCATGCTTTGGTACAAGTGCT
NLS-GFP	CCTAAGAAGAAGCGTAAGGAGAGCGA CGAGAGCG	CTATTCTTCACCGGCATCTG
	**5'Splint Oligo**	**3'Splint Oligo**
FOXA1	CCTTCCATCTTCACAGTTCCTAACATG GTGGCTCTTATATTTCTTCTT	CCCGCAGAAGGCAGCCTAGGAAGTGT TTAGGACG
CEBPA	CGTAGAAGTCGGCCGACTCCATGGTG GCTCTTATATTTCTTCTT	CCCGCAGAAGGCAGCTCACGCGCAGT TGCCCAT
FOXA2	AGCATACTGGAAGCCGAGTGCATGGT GGCTCTTATATTTCTTCTT	CCCGCAGAAGGCAGCTTAAGAGGAGT TCATAATGGGC
FOXA3	CCATCTTCACTGAGCCCAGCATGGTGG CTCTTATATTTCTTCTT	CCCGCAGAAGGCAGCCTAGGATGCAT TAAGCAAAGAG
GATA4	CCATGGCCAAGCTCTGATACATGGTG GCTCTTATATTTCTTCTT	CCCGCAGAAGGCAGCTTACGCAGTGA TTATGTCCCC
GATA6	CCGCCGTCAGTCAAGGCCATGGTGGC TCTTATATTTCTTCTT	CCCGCAGAAGGCAGCTCAGGCCAGGG CCAGGG
HHEX	CGGGCCCCACGCCCATGACCATGGTG GCTCTTATATTTCTTCTT	CCCGCAGAAGGCAGCTCATCCAGCATT AAAATAGC
HNF1A	GCAGCTGGCTCAGTTTAGAAACCATG GTGGCTCTTATATTTCTTCTT	CCCGCAGAAGGCAGCTTACTGGGAGG AAGAGGC
HNF1B	CGACGTGAGCTTGGACACCATGGTGG CTCTTATATTTCTTCTT	CCCGCAGAAGGCAGCCCAGGCTTGTA GAGGACACT
HNF4A	CGAGGGTTTTGGAGAGTCGCATGGTG GCTCTTATATTTCTTCTT	CCCGCAGAAGGCAGCCTAGATAACTT CCTGCTTGG
HNF6A	ATGGTCAGCTGCGCGTTCATGGTGGC TCTTATATTTCTTCTT	CCCGCAGAAGGCAGCTCATGCTTTGG TACAAGTGC
NLS-GFP	CTCTCCTTACGCTTCTTCTTAGGCATG GTGGCTCTTATATTTCTTCTT	CCCGCAGAAGGCAGCCTATTCTTCACC GGCATCTG
	**Forward Tailing Primer**	**Reverse Tailing Primer**
Tailing	TTGGACCCTCGTACAGAAGC	T_{120} CTTCCTACTCAGGCTTTATTCAAA
	**5'UTR**	**3'UTR**
UTRs	TTGGACCCTCGTACAGAAGCTAATAC GACTCACTATAGGGAAATAAGAGAGA AAAGAAGAGTAAGAAGAAATATAAGA GCCACCATG	GCTGCCTTCTGCGGGGCTTGCCTTCTG GCCATGCCCTTCTTCTCTCCCTTGCAC CTGTACCTCTTGGTCTTTGAATAAAGC CTGAGTAGGAAGTGAGGGTCTAGAAC TAGTGTCGACGC
