# Supplementary material for: Direct Reprogramming of Human Fibroblasts to Hepatocyte-Like Cells by Synthetic Modified mRNAs
Source: PLoS One. 2014 Jun 25;9(6):e100134. doi: 10.1371/journal.pone.0100134 (PMC4070971; doi:10.1371/journal.pone.0100134)
Supplement: Table S3 — List of Taqman assays used for qPCR gene expression. (PDF) [file pone.0100134.s009.pdf]

Table S3. List of Taqman assays used for qPCR gene expression.

Gene	Assay ID
ABCB1	Hs01067802_m1
ABCB11	Hs00184824_m1
ABCG2	Hs01053790_m1
AFM	Hs00265717_m1
AFP	Hs01040601_m1
AFP	Hs01040601_m1
ALB	Hs00910225_m1
ALB	Hs00609411_m1
B2M	Hs00984230_m1
CD106	Hs01003372_m1
CD13	Hs00174265_m1
CD133	Hs01009250_m1
CD26	Hs00175210_m1
CD29	Hs00559595_m1
CD36	Hs00169627_m1
CD44	Hs01075861_m1
CD73	Hs00159686_m1
CD9	Hs00233521_m1
CD90	Hs00174816_m1
CDH2	Hs00983056_m1
CXCR4	Hs00237052_m1
CYP1A2	Hs00167927_m1
CYP2C19	Hs00426380_m1
CYP2C8	Hs02383390_s1
CYP2C9	Hs00426397_m1
CYP2D6	Hs00164385_m1
CYP2E1	Hs00559368_m1
CYP3A4	Hs00256159_m1
CYP3A7	Hs00426361_m1
CYP7A1	Hs00167982_m1
DES	Hs00157258_m1
DLK1	Hs00171584_m1
EGFR	Hs00193306_m1
FABP1	Hs00155026_m1
FGFR4	Hs00242558_m1
FOXA1	Hs04187555_m1
FOXA2	Hs00232764_m1
FOXA3	Hs00270130_m1
GAPDH	Hs03929097_g1
GATA4	Hs00171403_m1
GPC3	Hs00170471_m1
GSTA1	Hs00275575_m1
HNF1A	Hs00167041_m1
HNF4A	Hs00230853_m1
ICAM1	Hs00164932_m1
IGF2	Hs01005970_m1
IL6R	Hs00794121_m1
KRT15	Hs00267035_m1
KRT18	Hs01941416_g1
KRT19	Hs00761767_s1
KRT8	Hs01630795_s1
MET	Hs00179845_m1
NNMT	Hs00196287_m1
OSMR	Hs00384276_m1
RPL19	Hs02338565_gH
SERPINA1	Hs01097800_m1
SERPINA3	Hs00153674_m1
SLCO1B3	Hs00251986_m1
SLCO2B1	Hs00200670_m1
TTR	Hs00174914_m1
UGT2B4	Hs02383831_s1
